# Selection of performance-tested young bulls and indirect responses in commercial beef cattle herds on pasture and in feedlots

**DOI:** 10.1186/s12711-016-0265-2

**Published:** 2016-11-09

**Authors:** Fernanda S. S. Raidan, Dalinne C. C. Santos, Mariana M. Moraes, Andresa E. M. Araújo, Henrique T. Ventura, José A. G. Bergmann, Eduardo M. Turra, Fabio L. B. Toral

**Affiliations:** 1 Departamento de Zootecnia, Escola de Veterinária, Universidade Federal de Minas Gerais, Belo Horizonte, MG 31270-901 Brazil; 2 School of Chemistry and Molecular Biosciences, The University of Queensland, 4072 Brisbane, QLD Australia; 3 Associação Brasileira dos Criadores de Zebu, Uberaba, MG 38022-330 Brazil

## Abstract

**Background:**

Central testing is used to select young bulls which are likely to contribute to increased net income of the commercial beef cattle herd. We present genetic parameters for growth and reproductive traits on performance-tested young bulls and commercial animals that are raised on pasture and in feedlots.

**Methods:**

Records on young bulls and heifers in performance tests or commercial herds were used. Genetic parameters for growth and reproductive traits were estimated. Correlated responses for commercial animals when selection was applied on performance-tested young bulls were computed.

**Results:**

The 90% highest posterior density (HPD90) intervals for heritabilities of final weight (FW), average daily gain (ADG) and scrotal circumference (SC) ranged from 0.41 to 0.49, 0.23 to 0.30 and 0.47 to 0.57, respectively, for performance-tested young bulls on pasture, from 0.45 to 0.60, 0.20 to 0.32 and 0.56 to 0.70, respectively, for performance-tested young bulls in feedlots, from 0.29 to 0.33, 0.14 to 0.18 and 0.35 to 0.45, respectively, for commercial animals on pasture, and from 0.24 to 0.44, 0.13 to 0.24 and 0.35 to 0.57 respectively, for commercial animals in feedlots. The HPD90 intervals for genetic correlations of FW, ADG and SC in performance-tested young bulls on pasture (feedlots) with FW, ADG and SC in commercial animals on pasture (feedlots) ranged from 0.86 to 0.96 (0.83 to 0.94), 0.78 to 0.90 (0.40 to 0.79) and from 0.92 to 0.97 (0.50 to 0.83), respectively. Age at first calving was genetically related to ADG (HPD90 interval = −0.48 to −0.06) and SC (HPD90 interval = −0.41 to −0.05) for performance-tested young bulls on pasture, however it was not related to ADG (HPD90 interval = −0.29 to 0.10) and SC (HPD90 interval = −0.35 to 0.13) for performance-tested young bulls in feedlots.

**Conclusions:**

Heritabilities for growth and SC are higher for performance-tested young bulls than for commercial animals. Evaluating and selecting for increased growth and SC on performance-tested young bulls is efficient to improve growth, SC and age at first calving in commercial animals. Evaluating and selecting performance-tested young bulls is more efficient for young bulls on pasture than in feedlots.

## Background

Central testing of beef cattle is used quite widely worldwide since the 1950s, especially in the United States and Canada [1], Europe [2] and Brazil [3]. The aim of central testing is to identify young bulls as parents of the next generation which are likely to contribute to increased net income of commercial herds. Young bulls need to be raised under uniform housing, feeding, management and data recording to more accurately estimate the genetic merit of each animal. Growth, carcass, feed efficiency and scrotal circumference are measured during the test or at the end of the test [4–8]. Performance tests can be conducted on pasture or in feedlots. Feeding costs (per day and total cost) for testing young bulls are lower on pasture than in feedlots. However, pasture performance tests take longer than feedlot performance tests [6, 8–12].

After individual testing, outstanding young bulls can be used for breeding, either with or without progeny test, or sold to cow-calf producers. Therefore, the impact of selection for improved economic traits in performance-tested young bulls on growth and reproductive traits of young bulls and heifers in commercial herds is of particular importance. The genetic correlations (±standard error) of average daily gain and mid-test body weight of performance-tested young bulls in feedlots with post-weaning weight (12 to 36 months of age) of commercial animals on pasture present moderate magnitude (0.33 ± 0.15 and 0.56 ± 0.14, respectively) [4]. However, genetic correlations of growth in performance-tested young bulls in feedlots with age at first calving in commercial herds are weak (0.21 ± 0.15 and −0.18 ± 0.13, respectively) [5]. Furthermore, genetic correlations between growth and reproductive traits in performance test and commercial herds both on pasture or in feedlot are unknown. Availability of such data would be useful to evaluate the efficiency of selection in performance tests for the improvement of economic traits in commercial herds and to determine the best environment to carry out performance tests of young bulls. Thus, our aim was to estimate genetic parameters for growth and reproductive traits in performance-tested young bulls and commercial young bulls and heifers on pasture and in feedlots. In addition, we analyzed the impact of selecting performance-tested young bulls for growth and scrotal circumference on growth and reproductive traits in young bulls and heifers in commercial herds, both on pasture and in feedlots.

## Methods

### Data

Approval by the ethics committee was not necessary for this study because the data were obtained from an existing database. We used records from official performance tests on growth traits and scrotal circumference (SC) of Nellore young bulls on pasture and in feedlots and records from a joint official performance recording scheme on growth and reproductive traits (SC and age at first calving, AFC) of young bulls and heifers. Performance records and pedigree information were provided by Associação Brasileira de Criadores de Zebu (ABCZ).

The performance of 33,013 animals was evaluated in 751 performance tests that were carried out from 2003 to 2012 in the North (Acre, Rondônia, Pará, and Tocantins), Northeast (Bahia and Maranhão), Central West (Goiás, Mato Grosso and Mato Grosso do Sul), Southeast (Espírito Santo, Minas Gerais and São Paulo) and South (Paraná and Rio Grande do Sul) regions of Brazil. Our study included 24,910 animals from 538 tests that were conducted on pasture and 8103 animals from 213 tests that were conducted in feedlots. Pasture tests lasted 294 days (70 days for adaptation and 224 days for testing) and feedlot tests lasted 168 days (56 days for adaptation and 112 days for testing). Animals were weighed at the beginning and end of the adaptation period and at the end of the testing period. The assessed traits included final weight (FW), average daily gain (ADG) and SC. ADG was calculated as the difference between body weight at the end of the testing period (WEndT) and body weight at the end of the adaptation period (WEndA), divided by the difference between age at the end of the testing period and age at the end of the adaptation period (AEndA). FW was calculated using the following equations FW = WEndA + [ADG × (550 − AEndA)] and FW = WEndA + [ADG × (426 − AEndA)] for performance-tested young bulls on pasture and in feedlots, respectively. The values 550 and 426 are the official standard final ages (in days) according to ABCZ. Individual records for each trait that exceeded the intervals given by the means of the performance tests plus or minus 3.5 standard deviations were excluded, and growth and SC records of animals from performance test on pasture and in feedlots that included less than 20 and 8 animals, respectively, were also excluded.

Performance records of commercial young bulls and heifers were from the official performance recording scheme of ABCZ for commercial purebred herds in Central West (Goiás, Mato Grosso and Mato Grosso do Sul) and Southeast (Minas Gerais and São Paulo) regions of Brazil. These records were collected from 2005 to 2010. The animals were weighed at weaning (from 145 to 265 days of age, mean age of 205 days) and at yearling (from 490 to 610 days of age, mean age of 550 days). The assessed traits included FW and ADG of young bulls and heifers, SC of young bulls, both on pasture and in feedlots, and AFC of heifers on pasture. ADG was calculated as the difference between body weight at yearling (YW) and body weight at weaning (WW), divided by the difference between age at yearling and age at weaning (AW). FW was calculated as follows: FW = WW + [ADG + (550 − AW)]. Individual records for each trait that exceeded the intervals given by the means of contemporary groups plus or minus 4 standard deviations were excluded, and animals from contemporary groups that included less than 10 animals were also excluded. Contemporary groups included animals from the same herd, year and month of birth, sex, and feeding regimen at weaning and yearling (on pasture with or without mineral supplementation or in feedlots). The levels of energy and/or protein supplementation were not available in the dataset, and the feeding regimen at yearling of animals that were fed with any type of energy and/or protein supplementation was considered as a feedlot. A total of 84,565 animals (from 4148 contemporary groups on pasture) and 4468 animals (from 266 contemporary groups in feedlots) were used in this study. Records on AFC were from heifers with growth records (FW and ADG) in the dataset, which originated from 540 contemporary groups on pasture. Heifers with AFC records represented 17.7% of the heifers with growth records. Summary statistics of these data are in Table 1 and the distributions of animals and sires across geographical regions are in Table 2.

**Table 1:** Summary statistics for growth and reproductive traits in performance-tested and commercial young bulls and heifers on pasture and in feedlots

Trait	N	Mean	SD	CV (%)
Performance test on pasture				
Final age (days)^a^	24,910	553.05	24.39	4.41
Final age (days)^b^	14,888	552.72	25.24	4.57
FW (kg)	24,910	350.35	53.09	15.15
ADG (kg/day)	24,910	0.54	0.16	29.63
SC (cm)	14,888	26.61	3.38	12.70
Commercial on pasture				
Final age (days)^a^	84,565	549.46	24.30	4.42
Final age (days)^b^	14,663	548.35	24.39	4.45
FW (kg)	84,565	312.54	58.05	18.57
ADG (kg/day)	84,565	0.36	0.14	38.89
SC (cm)	14,663	25.91	3.67	14.16
AFC (days)	8060	1164.83	180.52	15.50
Performance test in feedlots				
Final age (days)^a^	8103	423.59	26.41	6.23
Final age (days)^b^	4676	420.73	28.01	6.66
FW (kg)	8103	371.65	57.13	15.37
ADG (kg/day)	8103	0.83	0.27	32.53
SC (cm)	4676	25.41	3.31	13.03
Commercial in feedlots				
Final age (days)^a^	4468	549.62	24.17	4.40
Final age (days)^b^	1365	548.59	24.16	4.40
FW (kg)	4468	389.41	71.41	18.34
ADG (kg/day)	4468	0.54	0.18	33.33
SC (cm)	1365	28.46	3.95	13.88

*N* number of records, *SD* standard deviation, *CV* coefficient of variation (in %), *FW* final weight, *ADG* average daily gain, *SC* scrotal circumference, *AFC* age at first calving

^a^Only for animals with FW and ADG data

^b^Only for animals with SC data

**Table 2:** Distribution of animals and sires across geographical regions

Trait	Animals					Sires					
	NO	NE	CW	SE	SO	NO	NE	CW	SE	SO	Total
Performance tests on pasture											
Growth	4874	1317	7816	9769	1134	672	288	903	901	120	2047
SC	3243	1094	4581	5413	557	480	236	571	579	72	1347
Commercial on pasture											
Growth	–	–	46,878	37,687	–	–	–	2136	1423	–	3021
SC	–	–	8090	6573	–	–	–	958	578	–	1313
AFC	–	–	4456	753	–	–	–	3604	510	–	1053
Performance tests in feedlots											
Growth	69	–	4307	3051	676	20	–	463	303	80	688
SC	69	–	3281	1288	38	20	–	369	170	10	469
Commercial in feedlots											
Growth	–	–	2458	2010	–	–	–	325	308	–	527
SC	–	–	760	605	–	–	–	146	133	–	227

*NO* north, *NE* northeast, *CW* central west, *SE* southeast, *SO* south, *Growth* includes final weight and average daily gain, *SC* scrotal circumference, *AFC* age at first calving

**Fig. 1:**
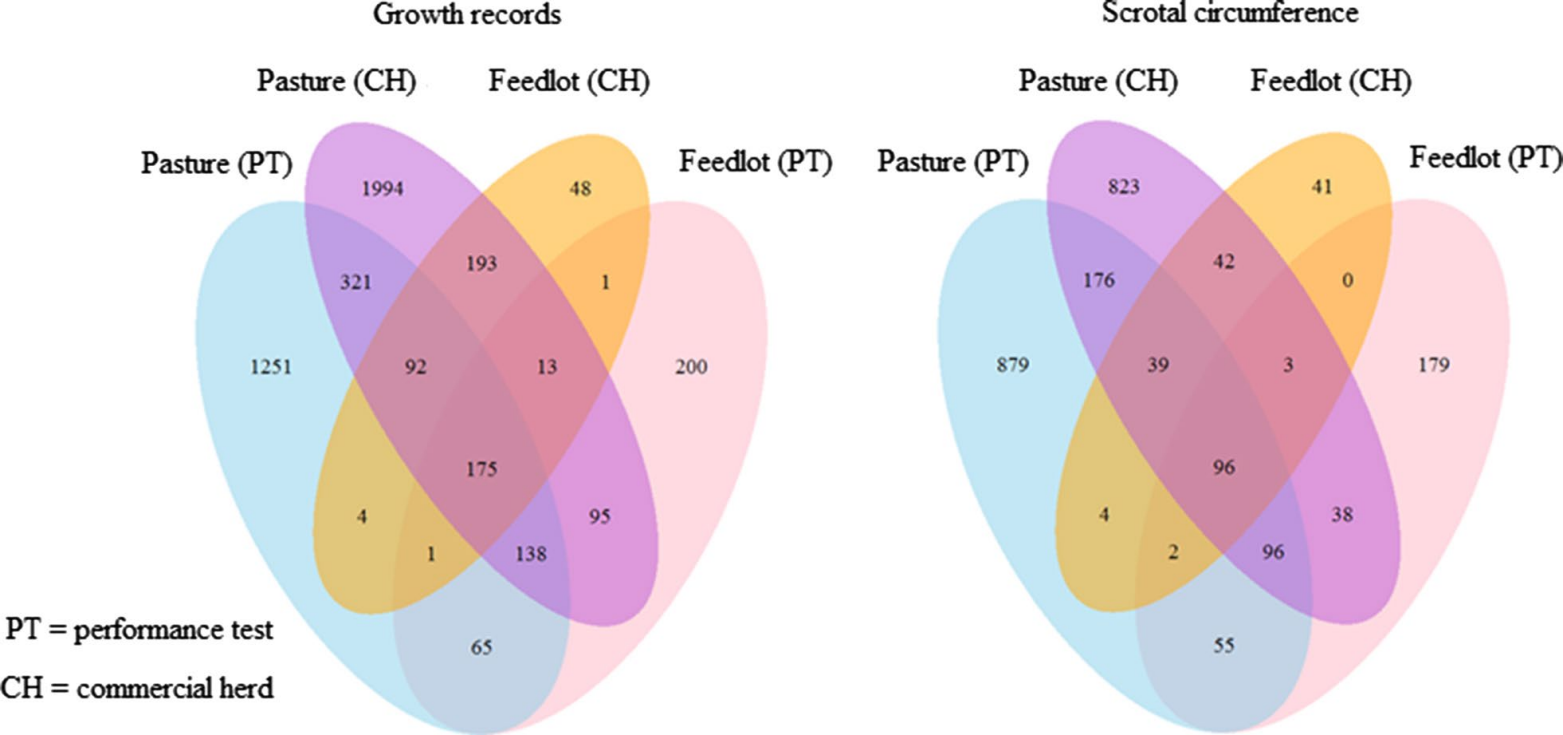
Number of sires with progeny records for growth and scrotal circumference across performance tests and commercial herds on pasture and in feedlots

The numerator relationship matrix considered pedigree data on 122,046 animals with records and ancestors of recorded animals, which resulted in 377,217 animals. The mean, minimum and maximum numbers of known generations for animals with at least one available record were 6.4, 1.5 and 8.9, respectively. The environmental connectedness through the use of common sires is shown in Fig. 1.

### Statistical analyses

Samples of the posterior distributions of the genetic parameters were obtained using a Bayesian approach and Gibbs sampler in multiple-trait analyses. The following general statistical model was used:

$$y_{hijk} = u_{h} + CG_{hj} + b_{{h_{\left( j \right)} }} \left( {A_{k} - \overline{{A_{j} }} } \right) + a_{hi} + e_{hijk} ,$$

where *y*_*hijk*_ is the observation for trait *h* on animal *i* in performance test (or contemporary group) *j* with final age *k*; *u*_*h*_ is the general constant present in each observation for trait *h*; *CG*_*hj*_ is the effect of performance test (or contemporary group) *j* for trait *h*; $b_{{h_{\left( j \right)} }}$ is the linear regression coefficient of final age for trait *h*, nested in the performance test (or contemporary group) *j*; *A*_*k*_ is the age *k*; $\overline{{A_{j} }}$ is the mean of the final ages of the animals from the contemporary group *j*; *a*_*hi*_ is the breeding value of animal *i* for trait *h*; and *e*_*hijk*_ is the residual effect for each observation. The effect of age was not included for AFC.

In matrix notation, the following general model was used in multiple-trait analyses:

$$\left[ {\begin{array}{*{20}c} {\mathop {{\mathbf{y}}_{1} }\limits_{\sim} } \\ {\mathop {{\mathbf{y}}_{2} }\limits_{\sim} } \\ \vdots \\ {\mathop {{\mathbf{y}}_{8} }\limits_{\sim} } \\ \end{array} } \right] = \left[ {\begin{array}{*{20}c} {{\mathbf{X}}_{1} } & {\varvec{\Phi}} & \cdots & {\varvec{\Phi}} \\ {\varvec{\Phi}} & {{\mathbf{X}}_{2} } & \cdots & {\varvec{\Phi}} \\ \vdots & \vdots & \ddots & \vdots \\ {\varvec{\Phi}} & {\varvec{\Phi}} & \cdots & {{\mathbf{X}}_{8} } \\ \end{array} } \right]\left[ {\begin{array}{*{20}c} {\mathop {{\varvec{\upbeta}}_{1} }\limits_{\sim} } \\ {\mathop {{\varvec{\upbeta}}_{2} }\limits_{\sim} } \\ \vdots \\ {\mathop {{\varvec{\upbeta}}_{8} }\limits_{\sim} } \\ \end{array} } \right] + \left[ {\begin{array}{*{20}c} {{\mathbf{Z}}_{1} } & {\varvec{\Phi}} & \cdots & {\varvec{\Phi}} \\ {\varvec{\Phi}} & {{\mathbf{Z}}_{2} } & \cdots & {\varvec{\Phi}} \\ \vdots & \vdots & \ddots & \vdots \\ {\varvec{\Phi}} & {\varvec{\Phi}} & \cdots & {{\mathbf{Z}}_{8} } \\ \end{array} } \right]\left[ {\begin{array}{*{20}c} {\mathop {{\mathbf{a}}_{1} }\limits_{\sim} } \\ {\mathop {{\mathbf{a}}_{2} }\limits_{\sim} } \\ \vdots \\ {\mathop {{\mathbf{a}}_{8} }\limits_{\sim} } \\ \end{array} } \right] + \left[ {\begin{array}{*{20}c} {\mathop {{\mathbf{e}}_{1} }\limits_{\sim} } \\ {\mathop {{\mathbf{e}}_{2} }\limits_{\sim} } \\ \vdots \\ {\mathop {{\mathbf{e}}_{8} }\limits_{\sim} } \\ \end{array} } \right],$$

where **y**_h_ is the vector of records for trait *h*, **X**_h_ is the incidence matrix of fixed effects; **β**_h_ is the vector of fixed effects, **Z**_h_ is the incidence matrix of random effects; **a**_h_ is the vector of breeding values for trait *h* and **e**_h_ is the vector of residuals for trait *h*. **Φ** is the symbol for an empty matrix. The indexes *h* are as follows: FW, ADG and SC in performance-tested animals on pasture or in feedlots were defined as trait 1, FW, ADG, SC, AFC in commercial animals on pasture were defined as traits 2, 3, 4 and 5, respectively, and FW, ADG and SC in commercial animals in feedlots were defined as traits 6, 7 and 8, respectively. Thereby, six multiple-trait analyses were carried out.

Flat prior distributions were assumed for fixed effects $$\left( {\left[ {\begin{array}{*{20}c} {\mathop {{\varvec{\upbeta}}_{1} }\limits_{\sim} } & {\mathop {{\varvec{\upbeta}}_{2} }\limits_{\sim} } & \cdots & {\mathop {{\varvec{\upbeta}}_{8} }\limits_{\sim} } \\ \end{array} } \right]^{\text{t}} } \right)$$, and normal distributions were assumed for random effects $$\left( {\left. {\left[ {\begin{array}{*{20}c} {\mathop {{\mathbf{a}}_{1} }\limits_{\sim} } & {\mathop {{\mathbf{a}}_{2} }\limits_{\sim} } & \cdots & {\mathop {{\mathbf{a}}_{8} }\limits_{\sim} } \\ \end{array} } \right]^{\text{t}} } \right|{\mathbf{G}}} \right)$$ and $$\left( {\left. {\left[ {\begin{array}{*{20}c} {\mathop {{\mathbf{e}}_{1} }\limits_{\sim} } & {\mathop {{\mathbf{e}}_{2} }\limits_{\sim} } & \cdots & {\mathop {{\mathbf{e}}_{8} }\limits_{\sim} } \\ \end{array} } \right]^{\text{t}} } \right|{\mathbf{R}}} \right)$$, whereas inverted Wishart distributions were assumed for (co)variance matrices (**G**_**0**_|**ν**_**a**_, **S**_**a**_) and (**R**|**ν**_**e**_, **S**_**e**_), where **G** = **G**_**0**_ ⊗ **A** represents the genetic (co)variance matrix and $${\mathbf{G}}_{0} = \left[ {\begin{array}{*{20}c} {\upsigma_{{{\text{a}}_{1} }}^{2} } & {\upsigma_{{{\text{a}}_{1} {\text{a}}_{2} }} } & \cdots & {\upsigma_{{{\text{a}}_{1} {\text{a}}_{8} }} } \\ {\upsigma_{{{\text{a}}_{1} {\text{a}}_{2} }} } & {\upsigma_{{{\text{a}}_{2} }}^{2} } & \cdots & {\upsigma_{{{\text{a}}_{2} {\text{a}}_{8} }} } \\ \vdots & \vdots & \ddots & \vdots \\ {\upsigma_{{{\text{a}}_{1} {\text{a}}_{8} }} } & {\upsigma_{{{\text{a}}_{2} {\text{a}}_{8} }} } & \cdots & {\upsigma_{{{\text{a}}_{8} }}^{2} } \\ \end{array} } \right]$$ represents the matrix of the genetic (co)variances between traits 1 to 8; σ_ah_^2^ represents the additive genetic variance for trait *h*; $\upsigma_{{{\text{a}}_{\text{h}} {\text{a}}_{{{\text{h}}^{{\prime }} }} }}$ represents the additive genetic covariance between traits *h* and *h*′; **R** = **R**_0_ ⊗ **A** represents the residual variance matrix;

$${\mathbf{R}}_{0} = \left[ {\begin{array}{*{20}c} {\upsigma_{{{\text{e}}_{1} }}^{2} } & 0 & 0 & 0 & 0 & 0 & 0 & 0 \\ 0 & {\upsigma_{{{\text{e}}_{2} }}^{2} } & {\upsigma_{{{\text{e}}_{2} {\text{e}}_{3} }} } & {\upsigma_{{{\text{e}}_{2} {\text{e}}_{4} }} } & {\upsigma_{{{\text{e}}_{2} {\text{e}}_{5} }} } & 0 & 0 & 0 \\ 0 & {\upsigma_{{{\text{e}}_{2} {\text{e}}_{3} }} } & {\upsigma_{{e_{3} }}^{2} } & {\upsigma_{{{\text{e}}_{3} {\text{e}}_{4} }} } & {\upsigma_{{{\text{e}}_{ 3} {\text{e}}_{5} }} } & 0 & 0 & 0 \\ 0 & {\upsigma_{{{\text{e}}_{2} {\text{e}}_{4} }} } & {\upsigma_{{{\text{e}}_{3} {\text{e}}_{4} }} } & {\upsigma_{{{\text{e}}_{4} }}^{2} } & 0 & 0 & 0 & 0 \\ 0 & {\upsigma_{{{\text{e}}_{2} {\text{e}}_{5} }} } & {\upsigma_{{{\text{e}}_{3} {\text{e}}_{5} }} } & 0 & {\upsigma_{{{\text{e}}_{5} }}^{2} } & 0 & 0 & 0 \\ 0 & 0 & 0 & 0 & 0 & {\upsigma_{{{\text{e}}_{6} }}^{2} } & {\upsigma_{{{\text{e}}_{6} {\text{e}}_{7} }} } & {\upsigma_{{{\text{e}}_{6} {\text{e}}_{8} }} } \\ 0 & 0 & 0 & 0 & 0 & {\upsigma_{{{\text{e}}_{6} {\text{e}}_{7} }} } & {\upsigma_{{{\text{e}}_{7} }}^{2} } & {\upsigma_{{{\text{e}}_{7} {\text{e}}_{8} }} } \\ 0 & 0 & 0 & 0 & 0 & {\upsigma_{{{\text{e}}_{6} {\text{e}}_{8} }} } & {\upsigma_{{{\text{e}}_{7} {\text{e}}_{8} }} } & {\upsigma_{{{\text{e}}_{8} }}^{2} } \\ \end{array} } \right]$$

represents the matrix of residual variance of traits 1 to 8; σ_eh_^2^ is the residual variance for trait *h*; $\sigma_{{{\text{e}}_{\text{h}} {\text{e}}_{\text{h'}} }}$ is the residual covariance between traits *h* and *h*′; **ν**_**a**_ and **ν**_**e**_ (degrees of freedom of the inverted Wishart distributions) and **S**_**a**_ and **S**_**e**_ (8 × 8 matrices of (co)variance components obtained from preliminary analyses) are the hyper-parameters of the inverted Wishart distributions of genetic and residual (co)variances; and the other terms are the same as those described above. The complete conditional posterior distributions are in Sorensen and Gianola [13].

Gibbs chains of 410,000 iterations were generated for each parameter, with a burn-in period of 10,000 iterations and a sampling interval of 200 iterations in the GIBBS1F90 program [14]. Gibbs chain size, burn-in period and sampling interval were those determined in previous analyses. The genetic and residual variances for FW, ADG, SC and AFC of commercial animals on pasture and FW, ADG and SC of commercial animals in feedlots that are shown in this paper were the means of 12,000 samples obtained in six multiple-trait analyses. Convergence diagnostics were performed by following Geweke’s [15] and Heidelberger and Welch’s [16] techniques and a visual analysis of the trace plots was performed by using the Bayesian Output Analysis [17] program in R software 3.2.3 [18].

Samples of posterior distributions for efficiency of correlated response (ECR), considering the same intensity of selection for traits in performance-tested and commercial animals, were obtained by the following equation available in Falconer and Mackay [19]:

$${\text{ECR}}_{{{\text{hh}}^{{\prime }} }} = \frac{{\Delta {\mathbf{G}}_{{{\text{hh}}^{{\prime }} }} }}{{\Delta {\mathbf{G}}_{\text{h}} }} = {\text{r}}_{{{\text{a}}_{{{\text{hh}}^{{\prime }} }} }} \frac{{{\text{h}}_{{{\text{h}}^{{\prime }} }} }}{{{\text{h}}_{\text{h}} }},$$

where $\Delta {\mathbf{G}}_{{{\text{hh}}^{{\prime }} }}$ is the expected genetic gain per generation for trait *h* in commercial animals when selection was applied for trait *h*′ in performance-tested animals; Δ**G**_h_ is the expected genetic gain per generation for trait *h* in commercial animals; *h*′ is the trait under selection in performance-tested animals; *h* is the indirectly selected trait in commercial animals; ${\text{r}}_{{{\text{a}}_{{{\text{hh}}^{{\prime }} }} }}$ is the genetic correlation between traits *h* and *h*′; and ${\text{h}}_{{{\text{h}}^{{\prime }} }}$ and h_h_ are the square roots of the heritabilities for traits *h*′ and *h*, respectively.

In addition to the analyses previously described, two multiple-trait analyses were performed in which FW or ADG of performance-tested animals on pasture were defined as trait 1, FW and ADG of male commercial animals on pasture were defined as traits 2 and 3, respectively, and FW, ADG and AFC of female commercial animals on pasture were defined as traits 4, 5 and 6, respectively. These analyses were performed to estimate the genetic correlations for the same trait between young bulls and heifers. Furthermore, we carried out another two analyses for the same traits measured on performance-tested and commercial animals in feedlots. A single-trait analysis for AFC was run to compare the results from single and multiple-trait analyses for this trait.

## Results

### Genetic variation of growth and reproductive traits

Posterior means and the 90% highest posterior density (HPD90) intervals of the variances and heritabilities for growth and reproductive traits in performance-tested and commercial young bulls and heifers are in Table 3. The posterior means of the additive genetic variances for FW and ADG were higher for performance-tested young bulls than for commercial animals on pasture or in feedlots (Table 3).

**Table 3:** Variance components for growth and reproductive traits in performance-tested and commercial young bulls and heifers on pasture and in feedlots

Trait	σ_a_^2^	σ_e_^2^	*h* ^2^
Performance test on pasture			
FW	421.03 (380.00; 461.80)	514.38 (487.00; 547.60)	0.45 (0.41; 0.49)
ADG	0.019 (0.016; 0.022)	0.053 (0.051; 0.055)	0.26 (0.23; 0.30)
SC	3.34 (2.94; 3.69)	3.05 (2.79; 3.33)	0.52 (0.47; 0.57)
Commercial on pasture			
FW	322.26 (295.70; 345.30)	721.84 (702.80; 739.90)	0.31 (0.29; 0.33)
M_FW	321.08 (281.90; 358.30)	887.12 (857.30; 916.10)	0.27 (0.24; 0.29)
F_FW	264.14 (238.10; 286.90)	604.12 (585.20; 623.30)	0.30 (0.27; 0.33)
ADG	0.010 (0.009; 0.011)	0.051 (0.050; 0.055)	0.16 (0.14; 0.18)
M_ADG	0.012 (0.011; 0.014)	0.058 (0.057; 0.060)	0.18 (0.15; 0.20)
F_ADG	0.009 (0.008; 0.010)	0.044 (0.042; 0.045)	0.17 (0.15; 0.20)
SC	2.58 (2.20; 2.91)	3.86 (3.59; 4.13)	0.40 (0.35; 0.45)
AFC	3.65 (1.93; 4.36)	15.50 (14.69; 16.91)	0.18 (0.10; 0.22)
AFC^a^	1.68 (1.20; 2.16)	16.96 (16.33; 17.57)	0.09 (0.06; 0.11)
Performance test in feedlots			
FW	756.70 (626.30; 895.80)	689.82 (590.40; 780.30)	0.52 (0.45; 0.60)
ADG	0.064 (0.048; 0.082)	0.181 (0.168; 0.195)	0.26 (0.20; 0.32)
SC	4.27 (3.64; 4.88)	2.49 (2.07; 2.97)	0.63 (0.56; 0.70)
Commercial in feedlots			
FW	426.53 (308.00; 586.90)	860.56 (749.80; 976.40)	0.33 (0.24; 0.44)
M_FW	355.59 (298.10; 432.20)	984.17 (915.50; 1060.00)	0.27 (0.22; 0.31)
F_FW	473.95 (319.40; 645.20)	687.18 (549.70; 803.20)	0.41 (0.28; 0.53)
ADG	0.015 (0.010; 0.019)	0.064 (0.060; 0.070)	0.19 (0.13; 0.24)
M_ADG	0.013 (0.008; 0.018)	0.069 (0.065; 0.075)	0.16 (0.09; 0.22)
F_ADG	0.013 (0.007; 0.018)	0.060 (0.054; 0.066)	0.17 (0.09; 0.23)
SC	3.62 (2.65; 4.63)	4.16 (3.39; 4.99)	0.46 (0.35; 0.57)

Lower and upper limits of the highest posterior density intervals with 90% of the samples are listed between brackets

Posterior means of *σ*_*a*_^*2*^ additive genetic variance, *σ*_*e*_^*2*^ residual variance, *h* ^2^ heritability, *FW* final weight, *M_FW* male FW, *F_FW* female FW, *ADG* average daily gain, *M_ADG* male ADG, *F_ADG* female ADG, *SC* scrotal circumference, *AFC* age at first calving

^a^Results from single trait analysis. Variances for AFC were multiplied by 10^−3^

The posterior means of the additive genetic variance for SC were higher for performance-tested young bulls on pasture than for commercial animals on pasture. However, the additive genetic variances for SC were similar between performance-tested young bulls in feedlots and animals in commercial herds in feedlots (Table 3), because of overlapping HDP90 intervals. In addition, residual variances for FW and SC were smaller for performance-tested young bulls than for commercial animals, and the posterior mean of the residual variance for ADG was higher for performance-tested animals in feedlots than for commercial animals in feedlots (Table 3). Estimated heritabilities were higher for traits in performance-tested young bulls than in commercial animals (Table 3).

The posterior means of the additive genetic and residual variances for FW and ADG were higher for males than for females in commercial herds on pasture and estimated residual variances for FW and ADG were higher for males than for females in commercial herds in feedlots (Table 3). Estimated heritabilities for FW and ADG were similar between males and females in commercial herds on pasture, those for FW were higher for females than for males in commercial herds in feedlots, but with overlapping HDP90 intervals and those for ADG were similar between males and females in commercial herds in feedlots (Table 3).

The additive genetic variance and heritability for AFC were lower in the single-trait than in the multiple-trait analyses (Table 3).

### Genetic correlations between male and female traits

Posterior means (and the lower and upper limits of the HDP90 intervals between brackets) of the genetic correlations between male and female FW and ADG in commercial herds on pasture were equal to 0.96 (0.94; 0.98) and 0.75 (0.58; 0.88), respectively. Genetic correlations between male and female FW and ADG in commercial herds in feedlots were equal to 0.96 (0.93; 0.99) and 0.74 (0.63; 0.85), respectively.

### Genetic correlations between performance test and commercial herds traits

Posterior means of the genetic correlations of FW, ADG and SC between performance-tested and commercial animals were positive (Table 4), which indicates that selection for either of these traits in performance-tested young bulls will result in improved growth and SC in commercial animals.

**Table 4:** Genetic correlation between growth and reproductive traits in performance-tested young bulls on pasture and feedlot with growth and reproductive traits in commercial young bulls and heifers on pasture and in feedlots

Traits	Performance-tested young bulls on pasture			Performance-tested young bulls in feedlots		
	FW	ADG	SC	FW	ADG	SC
FW^a^	0.91 (0.86; 0.96)	0.63 (0.54; 0.78)	0.37 (0.27; 0.46)	0.87 (0.82; 0.91)	0.60 (0.47; 0.71)	0.53 (0.44; 0.63)
ADG^a^	0.69 (0.62; 0.76)	0.84 (0.78; 0.90)	0.27 (0.18; 0.37)	0.40 (0.30; 0.51)	0.39 (0.27; 0.52)	0.24 (0.11; 0.36)
SC^a^	0.32 (0.22; 0.40)	0.27 (0.16; 0.37)	0.94 (0.92; 0.97)	0.28 (0.16; 0.40)	0.17 (0.00; 0.33)	0.80 (0.73; 0.88)
AFC^a^	−0.19 (−0.38; 0.09)	−0.26 (−0.48; −0.06)	−0.23 (−0.41; −0.05)	0.02 (−0.17; 0.18)	−0.06 (−0.29; 0.10)	−0.11 (−0.35; 0.13)
FW^b^	0.66 (0.54; 0.78)	0.33 (0.17; 0.54)	0.25 (0.10; 0.38)	0.88 (0.83; 0.94)	0.65 (0.52; 0.77)	0.33 (0.18; 0.47)
ADG^b^	0.54 (0.38; 0.71)	0.39 (0.23; 0.56)	0.23 (0.03; 0.42)	0.72 (0.60; 0.85)	0.58 (0.40; 0.79)	0.26 (0.12; 0.40)
SC^b^	0.12 (−0.10; 0.34)	0.12 (−0.10; 0.28)	0.73 (0.63; 0.83)	0.49 (0.38; 0.61)	0.56 (0.45; 0.70)	0.67 (0.50; 0.83)

Lower and upper limits of the highest posterior density intervals with 90% of the samples are listed between brackets

*FW* final weight, *ADG* average daily gain, *SC* scrotal circumference, *AFC* age at first calving

^a^FW, ADG, SC and AFC in commercial young bulls and heifers on pasture

^b^FW, ADG and SC in commercial young bulls and heifers in feedlots

The posterior mean of the genetic correlation between FW in performance-tested young bulls on pasture and FW in commercial animals on pasture was higher than the genetic correlation between FW in performance-tested young bulls on pasture and FW in commercial animals in feedlots (Table 4). The same results were observed for ADG and SC (Table 4). These differences were not observed for genetic correlations of FW, ADG and SC between performance-tested young bulls in feedlots and commercial animals on pasture or in feedlots (Table 4).

Genetic correlations of ADG and SC in performance-tested young bulls on pasture with AFC in heifers on pasture were negative (Table 4). However, genetic correlations of FW in performance-tested young bulls on pasture and of FW, ADG and SC in performance-tested young bulls in feedlots with AFC were almost zero (Table 4). Thus, selection for ADG and SC in performance-tested young bulls on pasture will result in decreased AFC in commercial heifers but selection for FW in performance-tested young bulls on pasture or growth and SC in performance-tested young bulls in feedlots will have no effect on AFC in commercial heifers on pasture.

### Efficiency of correlated responses

Table 5 presents the efficiencies of correlated responses for FW, ADG, SC and AFC in commercial animals when FW, ADG and SC were selected in performance-tested young bulls.

**Table 5:** Efficiency of correlated responses for growth and reproductive traits in commercial young bulls and heifers on pasture and in feedlots when the selection is applied for increased growth and reproductive traits in performance-tested young bulls on pasture and in feedlots

Traits	Performance-tested young bulls on pasture			Performance-tested young bulls in feedlots		
	FW	ADG	SC	FW	ADG	SC
FW^a^	1.10 (1.03; 1.19)	0.58 (0.48; 0.68)	0.48 (0.35; 0.60)	1.12 (1.03; 1.22)	0.55 (0.43; 0.67)	0.74 (0.60; 0.90)
ADG^a^	1.16 (1.00; 1.13)	1.08 (0.94; 1.19)	0.49 (0.32; 0.67)	0.71 (0.54; 0.89)	0.50 (0.34; 0.68)	0.46 (0.24; 0.72)
SC^a^	0.34 (0.24; 0.43)	0.22 (0.13; 0.31)	1.08 (1.01; 1.16)	0.32 (0.17; 0.44)	0.14 (0.02; 0.27)	1.00 (0.90; 1.13)
AFC^a^	−0.33 (−0.68; 0.44)	−0.33 (−0.63; −0.03)	−0.44 (−0.85; −0.05)	0.04 (−0.33; 0.31)	−0.07 (−0.36; 0.14)	−0.20 (−0.73; 0.20)
FW^b^	0.78 (0.47; 0.99)	0.30 (0.09; 0.52)	0.32 (0.16; 0.49)	1.11 (0.98; 1.25)	0.59 (0.46; 0.75)	0.46 (0.24; 0.65)
ADG^b^	0.84 (0.56; 1.19)	0.47 (0.26; 0.71)	0.44 (0.06; 0.81)	1.25 (1.01; 1.53)	0.70 (0.38; 0.95)	0.50 (0.20; 0.84)
SC^b^	0.12 (−0.09; 0.32)	0.09 (−0.06; 0.22)	0.78 (0.64; 0.96)	0.50 (0.37; 0.65)	0.41 (0.29; 0.53)	0.76 (0.50; 0.99)

Lower and upper limits of the highest posterior density intervals with 90% of the samples are listed between brackets

*FW* final weight, *ADG* average daily gain, *SC* scrotal circumference, *AFC* age at first calving

^a^FW, ADG, SC and AFC in commercial young bulls and heifers on pasture

^b^FW, ADG and SC in commercial young bulls and heifers in feedlots

Correlated responses for (1) FW, ADG or SC in commercial animals on pasture when FW, ADG or SC were selected in performance-tested young bulls on pasture were similar or higher than the direct responses for FW, ADG or SC in commercial animals on pasture, respectively; (2) FW in commercial animals (on pasture or in feedlots) when FW was selected in performance-tested young bulls in feedlots were similar or higher than the direct responses for FW in commercial animals (on pasture or in feedlots); (3) SC in commercial animals on pasture when SC was selected in performance-tested young bulls in feedlots were similar to the direct response for SC in commercial animals on pasture; (4) ADG in commercial animals in feedlots when ADG was selected in performance-tested young bulls on pasture or in feedlots were similar; and (5) SC in commercial animals in feedlots when SC was selected in performance-tested young bulls on pasture or in feedlots were also similar.

## Discussion

### Genetic variation of growth and reproductive traits

The heritabilities, genetic correlations and response to selection for growth and SC in performance-tested young bulls on pasture and in feedlots were presented and discussed previously [8]. The results about these genetic parameters in commercial animals on pasture and in feedlots are quite similar to those presented in Raidan et al. [8]. In most cases, response to selection will be greater for animals in feedlots than on pasture (if selection intensities are the same) because feeding conditions are better and variances are larger for animals in feedlots than on pasture [8, 20, 21].

Genetic variances and heritabilities for growth and SC were higher for performance-tested young bulls than for commercial animals (Table 3). This higher genetic variance for performance-tested animals (except for FW and SC for animals in feedlots because there is difference in the final age between performance-tested and commercial animals) might be a consequence of overall conditions being better and of phenotypic means being higher in performance tests. In general, the environmental conditions (nutrition, sanitary management, etc.) are better for performance-tested young bulls than for commercial animals and they could be responsible for differences in the mean of each trait and in the expression of genetic differences [20, 21]. In addition, temporary random effects in performance testing are lower than in commercial herds because the changes in management conditions are less frequent, and the process of data recording is stricter in performance tests than in commercial conditions [12]. Moreover, the number of young bulls in each performance test was larger than the number of animals in each contemporary group of the commercial herds, which contributes to reduce the error associated with the estimation of systematic effects that are included in the statistical models. The residual variance for ADG is larger for performance-tested young bulls in feedlots than for commercial animals in feedlots because the mean ADG is more than 50% greater in performance tests in feedlots than in performance tests on pasture or in commercial herds on pasture and in feedlots.

AFC records probably originated from a selected group of heifers because the females that have a low weaning weight could have been culled at weaning and some heifers with a low body weight at yearling did not get pregnant during the first breeding season. Thus, the lowest posterior means of genetic variances and heritabilities were obtained from the single-trait analyses. However, the multiple-trait analyses were effective in reducing the bias from selection, as previously stated by Schaeffer [22]. In addition, the posterior mean of the heritability for AFC of commercial animals on pasture obtained from the multiple-trait analysis was similar to the mean heritability of 0.17 obtained from three different samples of Nellore heifers [23–25].

### Genetic correlations between male and female traits

Posterior means of heritabilities for growth traits were similar between males and females and genetic correlations between male and female growth traits were high (>0.74). These results agree with those of Garrick et al. [26], Rodríguez-Almeida et al. [27] and Van Vleck and Cundiff [28]. A large fraction of the additive genes for growth traits has the same effect with regard to controlling variation in each sex [26], and there is no evidence of genotype X sex interaction in commercial herds.

### Genetic correlations between performance test and commercial herd traits

The genetic correlation between the same trait in different environments has been one of the parameters used to indicate the existence of genotype X environment interaction. Falconer [29] suggested that a genetic correlation between the same trait in different environments lower than 1 is an evidence of genotype X environment interaction. In addition, James [30] and Mulder et al. [31] showed that it is important to have environment-specific breeding programs of progeny testing when the genetic correlations between the same trait in different environments are smaller than the thresholds of 0.70 and 0.61, respectively.

The genetic correlations between the same traits measured in performance-tested animals or in commercial herds were lower than 1, however the upper limits of the HDP90 intervals were higher than 0.79 (Table 4). Therefore, there is no practical effect of genotype X environment interaction for growth and SC of performance-tested and commercial beef cattle. In addition, heritabilities for traits of performance-tested young bulls were higher than heritabilities for the same traits in commercial animals (Table 3). Moreover, a combination of strong genetic correlation between direct and indirect selected traits and higher heritabilities for indirect traits suggest that indirect selection in performance tests is as efficient as direct selection in commercial herds.

Selection for increased ADG and SC in performance-tested young bulls on pasture will result in reduced AFC in commercial females on pasture. In the literature, estimates of genetic correlations between ADG and AFC range from −0.38 to −0.32 [23, 32] and between SC (at 12 or 18 months of age) and AFC from −0.42 to −0.22 [32, 33]. These results indicate that genes related to ADG and SC could also be related to AFC. In fact, at least one single nucleotide polymorphism (SNP) within the region between 78.85 and 79.85 Mb on chromosome 10, and one SNP within the region between 23.4 and 33.85 Mb on chromosome 14 have been reported to affect both SC and AFC in Nellore cattle [34, 35].

The posterior mean of the genetic correlation between FW in performance-tested young bulls on pasture and AFC was negative (Table 4), but the HDP90 interval included zero, which means that this genetic correlation is not different of zero. The genetic correlation between growth traits in performance-tested young bulls and commercial young bulls and heifers on pasture was sufficiently high to consider these traits in different environments as the same trait. The results in Table 4 suggest that AFC is more strongly correlated with ADG than with FW. The relationships between growth rate, age and live weight at puberty are very complex and it is virtually impossible to separate the effects of growth rate per se from those of live weight and/or age [36]. However, the genetic correlations of ADG and maturation rate with AFC (−0.32 and −0.83, respectively) are stronger than the genetic correlations of FW and weight at maturity with AFC (−0.26 and 0.52, respectively) [23, 37]. In addition, the selection for high growth rate results in a younger and heavier population at puberty [38]. A high growth rate before puberty would involve a considerably higher rate of accumulation of adipose tissue than a low growth rate [36], and this change in body composition can be an effective trigger for puberty [38]. However, the control of reproduction involves a wide variety of interacting mechanisms and it is unlikely that there is only one mechanism involved in the onset of puberty. In addition, the evidence for a relationship between body composition and puberty is not sufficient. A genetic correlation of −0.29 for fat trim from one-half carcass with age at puberty was reported in *Bos taurus* crossbred animals [39], a genetic correlation (±standard error) of 0.13 ± 0.09 for intramuscular fat percentage with heifer pregnancy was reported in the Red Angus breed [40], and an estimated genetic correlation of 0.11 of backfat thickness at 18 months with age at first calving was reported in Nellore breed with a HDP95 interval that ranged from −0.10 to 0.28 [41]. Moreover, based on these results [39–41], differences between breeds might be involved in accumulation of adipose tissue and onset of puberty.

Selection for increased FW, ADG and SC in performance-tested young bulls in feedlots will not change AFC (Tables 4, 5). The estimated genetic correlations of mid-test body weight and ADG in performance-tested young bulls in feedlots with AFC were equal to −0.18 ± 0.13 and 0.21 ± 0.15, respectively [5]. The large standard errors associated with these genetic correlations made it difficult to reach definitive conclusions on the implication of selection for increased growth in performance-tested young bulls in feedlots on AFC. However, the results of the selection experiment presented by Mercadante et al. [42] confirmed that a genetic correlation of almost 0 was found between FW in performance-tested young bulls in feedlots (378 days of age) and days to calving of the first mating, an indicative trait of AFC [43], in Nellore cattle. Similar results were observed for the Angus breed in Australia [44, 45]. Mercadante et al. [42] estimated significant genetic trends of 1.78 ± 0.20 and 2.39 ± 0.20 kg/year for FW and non-significant genetic trends of 0.03 ± 0.16 and 0.19 ± 0.17 days/year for days to calving of the first mating in two lines that were selected for increased FW, respectively. Later, Monteiro et al. [46] showed that selection for increased FW had no effect either on the development of the ovaries and the endometrium or the onset of puberty at 24 months of age in heifers. The selection for increased growth in performance-tested young bulls in feedlots will not change AFC in commercial heifers.

As stated above, AFC is more strongly correlated with ADG than with FW, but only a moderate genetic correlation between ADG of performance-tested young bulls in feedlots and ADG of commercial young bulls and heifers on pasture was observed (0.39, Table 4). Consequently, ADG of performance-tested young bulls in feedlots is not an efficient selection criterion for indirect improvement of ADG and AFC in commercial heifers on pasture.

Genetic correlations of ADG and FW between performance-tested young bulls on pasture (0.74) and in feedlots (0.67) are high [47], but the selection for one or the other had different consequences in commercial herds. Heritability of FW was higher than that of ADG (Table 3) and changes in FW or ADG were obtained in commercial animals when selection is for FW or ADG in performance-tested young bulls (Tables 4, 5), but selection for increased ADG will result in reduced AFC whereas selection for increased FW will not. FW is more correlated to body weight at the beginning of performance tests than ADG [6, 48], and currently there is no limit for differences in body weight at the beginning of performance tests. Consequently, FW is more affected by body weight at the beginning of the test and herd-of-origin effects than ADG. FW might be more correlated to adult body weight than ADG and increased adult body weight will result in increased energy requirements for the maintenance of cows [49]. These results suggest that ADG is better than FW as a post-weaning selection criterion.

### Correlated responses and implications for breeding

Performance testing can be used as a tool to evaluate and select bulls for commercial herds. Furthermore, the results obtained in our study and those obtained by Falconer [50] and Mascioli [51] show that pasture, compared to feedlot, is the best environment for the evaluation and selection of Nellore young bulls. Selection will be more efficient in an environment that allows the maximum expression of the genetic differences [8, 20, 21]. However, Falconer and Latyszewski [52] showed that the improvement obtained by selecting for growth traits on a high plane of nutrition did not carry over when the animals were transferred to a low plane of nutrition, but the improvement made on the low plane of nutrition was retained when the animals were transferred to a high plane of nutrition. Falconer [50] obtained direct and correlated responses for growth traits in mice on two planes of nutrition. The animals selected on a low plane of nutrition were heavier, had less fat and more protein, and females were better dams than those selected on the high plane of nutrition when the two groups were raised on the high plane of nutrition. Thus, selection should be made under the conditions that are the least favorable for the expression of the trait. This author observed the following differences in carcass composition: mice for which growth had been increased by selection on the low plane of nutrition were leaner than those for which growth had been increased by selection on the high plane of nutrition. These results indicate that increases in growth traits of mice on a high or low plane of nutrition were reached by using different physiological pathways [50].

Mascioli [51] conducted individual performance and progeny tests of Canchim young bulls on pasture and in feedlots. These bulls were ranked as superior, intermediate and inferior according to their FW in individual performance tests on pasture or in feedlots (approximately 400 days old). After individual performance tests, the bulls were submitted to progeny tests and their progenies were raised on pasture and in feedlots (progeny test). There is no effect of feedlot performance tests bull’s rank (superior, intermediate and inferior) on weaning weight and post-weaning growth of their progeny. However, the bull’s progeny that were ranked as superior on pasture performance test were heavier than other classes (intermediate and inferior) for birth weight, weaning weight and weight at 12 months. Mascioli [51] concluded that the selection of Canchim young bulls in favorable environments (feedlots) did not produce the same response to selection in restricted environments (pasture). Similarly, the results presented in Table 5 support the hypothesis that selection for ADG and SC of performance-tested animals on pasture is better than selection for ADG and SC of performance-tested animals in feedlots to improve the means of growth and reproductive traits in commercial animals on pasture or in feedlots.

## Conclusions

Heritabilities for growth and scrotal circumference are higher in performance-tested young bulls than in commercial young bulls and heifers, whereas the correlations between the same traits expressed in the different environments are high, implying that indirect selection based on performance test is efficient. Evaluation and selection for increased growth and scrotal circumference on performance-tested young bulls are efficient to improve growth, scrotal circumference and age at first calving in commercial animals. Average daily gain is a better post-weaning selection criterion than final weight in performance tests. Evaluating and selecting performance-tested young bulls is more efficient for animals on pasture than in feedlots.
